# Small but visible: Predicting rare bryophyte distribution and richness patterns using remote sensing-based ensembles of small models

**DOI:** 10.1371/journal.pone.0260543

**Published:** 2022-01-06

**Authors:** Carlos Cerrejón, Osvaldo Valeria, Jesús Muñoz, Nicole J. Fenton

**Affiliations:** 1 Institut de recherche sur les forêts, Université du Québec en Abitibi-Témiscamingue, boul. de l’Université, Rouyn-Noranda, Québec, Canada; 2 Hémera Centro de Observación de la Tierra, Escuela de Ingeniería Forestal, Facultad de Ciencias, Universidad Mayor, Huechuraba, Santiago, Chile; 3 Real Jardín Botánico (RJB-CSIC), Madrid, España; Instituto Federal de Educacao Ciencia e Tecnologia Goiano - Campus Urutai, BRAZIL

## Abstract

In Canadian boreal forests, bryophytes represent an essential component of biodiversity and play a significant role in ecosystem functioning. Despite their ecological importance and sensitivity to disturbances, bryophytes are overlooked in conservation strategies due to knowledge gaps on their distribution, which is known as the Wallacean shortfall. Rare species deserve priority attention in conservation as they are at a high risk of extinction. This study aims to elaborate predictive models of rare bryophyte species in Canadian boreal forests using remote sensing-derived predictors in an Ensemble of Small Models (ESMs) framework. We hypothesize that high ESMs-based prediction accuracy can be achieved for rare bryophyte species despite their low number of occurrences. We also assess if there is a spatial correspondence between rare and overall bryophyte richness patterns. The study area is located in western Quebec and covers 72,292 km^2^. We selected 52 bryophyte species with <30 occurrences from a presence-only database (214 species, 389 plots in total). ESMs were built from Random Forest and Maxent techniques using remote sensing-derived predictors related to topography and vegetation. Lee’s L statistic was used to assess and map the spatial relationship between rare and overall bryophyte richness patterns. ESMs yielded poor to excellent prediction accuracy (AUC > 0.5) for 73% of the modeled species, with AUC values > 0.8 for 19 species, which confirmed our hypothesis. In fact, ESMs provided better predictions for the rarest bryophytes. Likewise, our study revealed a spatial concordance between rare and overall bryophyte richness patterns in different regions of the study area, which have important implications for conservation planning. This study demonstrates the potential of remote sensing for assessing and making predictions on inconspicuous and rare species across the landscape and lays the basis for the eventual inclusion of bryophytes into sustainable development planning.

## 1. Introduction

Canadian boreal forests represent 24% of the world’s boreal forest [1]. In these forests, anthropogenic disturbances pose serious threats for boreal flora [2, 3]. This is particularly true for sensitive plant species such as bryophytes, which have been recognized as reliable indicators of environmental changes [4–6]. Bryophytes are key constituents of biodiversity in Canadian boreal forests, promoting species richness [7, 8] and supporting important ecosystem functions [8–10].

Forest management pressure is however affecting bryophyte diversity and community composition in the boreal biome, either through direct species removal or by altering habitat conditions originally suitable for bryophytes [11]. Forestry practices are also reducing the ecological continuity of forests, jeopardizing the recolonization processes after disturbance events [4, 12]. Highly habitat-specific and/or dispersal-limited bryophyte species harbored by old-growth boreal forests may therefore be at risk [12]. Despite their ecological importance and sensitivity to disturbances, bryophytes are part of the vast unseen biodiversity that is currently ignored in most conservation plans [13, 14].

Less known and represented in natural history collections than other groups such as birds, mammals or flowering plants, the large contribution of inconspicuous taxonomic groups to diversity is difficult to assess, and thus commonly operationalized using diversity measures of these other groups as surrogates [15, 16]. However, these better-known taxonomic groups are poor surrogates for highly diverse but less showy or studied taxa [17]. Including inconspicuous species groups, such as bryophytes (e.g. [18]), representativeness in systematic conservation planning assessments would lead to more robust conservation measures [19].

From a conservation perspective, rare species deserve priority attention as they are at a high risk of extinction [20, 21]. However, because of their own nature, many rare species of unseen biodiversity groups [19] suffer from a lack of information on environmental requirements or their distribution [22, 23]. Species Distribution Models (SDMs), which allow to quantify the statistical relationships between species observations and environmental conditions from known locations, can provide useful tools for assessing ecological preferences of rare species or predicting their distributions [24, 25]. More precisely, SDM-based predictions are achieved by using the relevant environmental conditions as proxies of species occurrence. However, the ability of traditional SDMs to predict rare species has been strongly limited by the number of occurrences available, with increases in prediction accuracy with increased sample size [26, 27]. Furthermore, modeling species with low prevalence often results in a high predictors/occurrences ratio, which can lead to model overfitting and reduced applicability to new data [28, 29]. Fortunately, recent advances in modeling techniques and approaches such as Ensembles of Small Models (ESMs) have been shown to provide robust predictions for rare plants [20, 28, 30]. ESMs are ensembles of bivariate models generated from all pairwise predictor combinations from a larger set of predictors [20, 28]. ESMs can produce more accurate predictions than traditional SDMs and reduce model overfitting for rare species [28]. In parallel, remote sensing (RS) offers a powerful tool to derive and integrate environmental information into SDMs and generate predictions on species distribution over large areas [18, 31, 32]. Although a considerable number of studies have successfully integrated RS predictors into SDMs [33–35], no study has generated ESMs using only RS predictors, nor has used this approach to generate SDMs of inconspicuous organisms such as bryophytes, much less of their rare species.

In this paper we use RS-derived predictors in an ESMs framework to produce predictive models of rare bryophyte species in Eastern Canadian boreal forests. Bryophyte rare species were selected based on their prevalence in the study area (<30 occurrences; [36]). This rare species selection approach was chosen because of the lack of knowledge on bryophytes related to their distribution, ecological preferences and abundance in the region [36], which make it difficult to apply more informative approaches such as multicriteria rare species classification methods (e.g. [37]). In fact, the most complete rare bryophyte species list published to date for the region used species’ prevalence as the only criterion for rare species classification [38–40]. It should be noted that rare bryophytes from [38–40] were not targeted here as their low prevalence (≤5 occurrences) greatly restricts the development of SDMs. We hypothesize that high ESMs-based prediction accuracy can be achieved for rare bryophyte species despite their low number of occurrences [28]. Our specific objectives are to assess i) if there is a relationship between the number of occurrences and the predictive performance of ESMs, ii) if the predictive performance of models varies by the modeled bryophyte guild (mosses, liverworts and sphagna), and iii) if there is a spatial relationship between the richness patterns of rare bryophyte species and overall bryophyte species both for bryophytes as a whole and at the guild level [18]. A total of 52 rare bryophyte species were targeted in the present study, including 33 mosses, 14 liverworts and 5 sphagna.

## 2. Materials and methods

### 2.1 Bryophyte field data set

**Fig 1:**
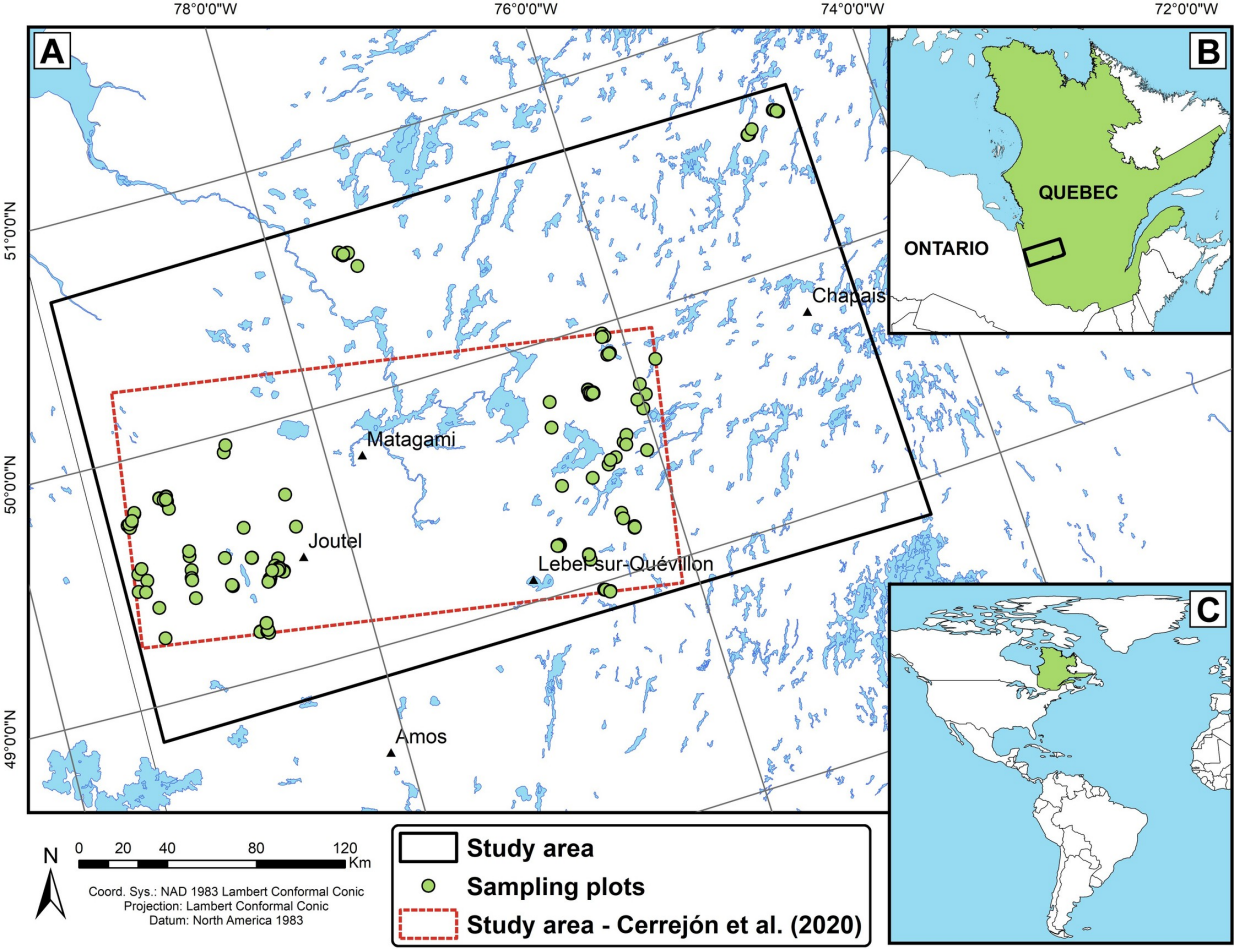
A: Study area and sampling plots (n = 389) in the boreal black spruce forest of western Quebec. B: Location of the study area within Quebec. C: Location of Quebec (eastern Canada).

We used a 389-plot database of presences-only including the field data from three studies previously conducted in our study area [41–43], which integrated young, mature and old-growth forests and both recent fires and cut-blocks. The study area of 72,292 km^2^ is located in the southwest of the Nord-du-Québec administrative region of western Quebec (48° 51’ to 50° 42’N and 74° 31’ to 79° 26’W; Fig 1), within the Black spruce–feathermoss forest bioclimatic domain [44]. Natural dynamics of these forests are primarily driven by stand-replacing fires, whose cycle has been estimated at 398 years after 1920 [45]. The region is characterized by a flat topography, dominance of poorly drained clay soils and a moderately humid and cold climate (927.8 mm annual precipitation and 1.0°C annual mean temperature) [46]. These conditions favor the accumulation of organic layer between fires, which is known as the paludification process [47, 48].

Bryophytes were collected following a “floristic habitat sampling” method, which consists in collecting all bryophytes found in all microhabitats within 5 x 10 m plots [49]. Rare bryophyte species were selected based on their prevalence within the study area (<30 occurrences) [36]. From an initial set of 214 species, 142 rare species were pre-selected, and among them, only those with a minimum of 5 occurrences were retained for modeling, since meaningful predictions can be achieved at this sample size [50–52]. A total of 52 rare bryophyte species (33 mosses, 14 liverworts and 5 sphagna; S1 Table in S1 Appendix) were finally selected for modeling (species occurrence coordinates are shown in S1 Table).

### 2.2 Remote sensing environmental predictors

The selection of RS-derived predictors was carried out based on their sensitivity to environmental factors known to influence bryophyte distribution, namely topography, canopy cover and structure, and vegetation and soil moisture [18, 53–55]. Climatic variables were not included due to their coarse spatial resolution (≥ 1 km) and low spatial variability across the study area (annual mean temperature and total precipitation with an approximate variability range of 1°C and 150 mm respectively [18]), which could lead us to overestimate the distribution of rare species. In addition, the climatic variability that could be integrated into the individual models of our rare species would be even more limited by the low number of available occurrences. It should be noted that climate variables also present lower reliability compared to RS variables at the scale of our study. This is because climatic variables are based on interpolation methods with high uncertainty, especially in northern latitudes where weather stations are scarce, while RS information is spatially continuous by nature. Therefore, we selected RS variables showing higher variability across the study area and capable of detecting changes in local conditions more closely related to bryophyte occurrence.

RS-derived environmental data were acquired using Google Earth Engine (GEE) [56]. The initial set of 6 predictors included topographic position index (TPI), 2-band enhanced vegetation index (EVI2), normalized difference water index (NDWI1), vegetation continuous fields (VCF), PALSAR HV/HH polarization index (PALSAR_HVHH), and bare soil index (BSI; see Table 1 for predictor descriptions). TPI was derived from the Shuttle Radar Topography Mission (SRTM) digital elevation model in ArcGIS v.10.5 [57] using an annulus neighborhood with inner and outer radius of 15 and 20 pixels, respectively. EVI2, NDWI1, and BSI predictors were derived from Sentinel-2 spectral bands. For each band, a mosaic was built from the images available for the summer season (July 1-August 31) between 2015–2019 to ensure homogeneity in the reflectance values [58]. Cloudy pixels were masked in all selected images using the Sentinel-2 QA60 band, which allows to identify pixels with opaque clouds and cirrus clouds. Mosaics were performed by applying the median of the overlapping pixel values. We chose EVI2 instead of EVI since EVI2 does not require the blue band, which is sensitive to the presence of residual clouds and aerosols [59]. VCF represents percent tree cover at 30 m resolution, after rescaling the 250 m MODIS VCF Tree Cover layer using circa-2010 and 2015 Landsat images and incorporating the MODIS Cropland Layer to improve accuracy in agricultural areas (https://catalog.data.gov/dataset/global-forest-cover-change-tree-cover-multi-year-global-30m-v003) [60]. The VCF predictor presented pixels (0.1% of the total) with missing values in the study area. PALSAR_HVHH was calculated as the ratio of HV-polarized to HH-polarized L-bands from the Advanced Land Observing Satellite (ALOS) Phased Arrayed L-band Synthetic Aperture Radar (SAR) [61]. HV-polarized and HH-polarized L-bands were averaged from yearly mosaics between 2015 and 2017. All predictors were generated and standardized at a 30 m spatial resolution (see Table 1 for original spatial resolutions). Pearson correlation coefficient was used to identify pairs of highly correlated predictors (|r|)> 0.7) from a set of 10,000 random background points. Only the NDWI1-BSI predictor pair showed a high correlation (r = -0.87). We retained NDWI1 which is sensitive to vegetation and soil moisture [62], since bryophytes are poikilohydric organisms whose distribution is highly dependent on available moisture [63, 64]. This resulted in a final set of 5 uncorrelated predictors to run the models (Table 1).

**Table 1:** Description of predictors by category and source.

Predictors	Description	Category	Data source	Source spatial resolution (m)
**TPI**	Topographic position index; relative elevation at one point compared to its surrounding environment (m); indicative of microclimate conditions [65]	Topography	SRTM	30 m
**EVI2**	2-band enhanced vegetation index (2.5 * (NIR—RED) / (NIR + 2.4 * RED + 1)); sensitive to photosynthetic active biomass [59, 66]	Vegetation	Sentinel-2	10 m
**NDWI1**	Normalized difference water index ((NIR–SWIR1) / (NIR + SWIR1)); sensitive to soil and vegetation moisture [62]	Vegetation	Sentinel-2	10 m; 20 m
**VCF**	Vegetation continuous fields; percent tree cover (%) [67]	Vegetation	MODIS	250 m
**PALSAR HVHH**	PALSAR HV/HH polarization index; indicative of forest structure [61]	Vegetation	ALOS PALSAR	25 m
BSI	Bare soil index ((SWIR1 + RED)–(NIR + BLUE) / (SWIR1 + RED) + (NIR + BLUE)); sensitive to bare soil areas and vegetated areas with different background [68]	Soil	Sentinel-2	10 m; 20 m

Uncorrelated predictors finally selected to model bryophyte distribution are shown in bold.

### 2.3 Modeling approach: Ensembles of small models

ESMs based on bivariate models were developed to spatially predict 52 rare bryophyte species (5–29 occurrences) using two modeling machine-learning techniques: Maxent [69] and Random Forest (RF) [70]. Both Maxent and RF techniques can provide robust predictions when few occurrences are available [50, 71, 72]. Maxent estimates the probability distribution for a given species by finding the probability distribution of maximum entropy according to a set of constraints representing the input known locations [69]. RF uses a bootstrap aggregation technique to provide mean predictions from a multitude of independent decision trees built from randomly selected subsamples from the training dataset [70]. A random subset of candidate predictors is assessed to split each node of each individual tree, selecting the predictor that provides the most information in each case [73].

**Fig 2:**
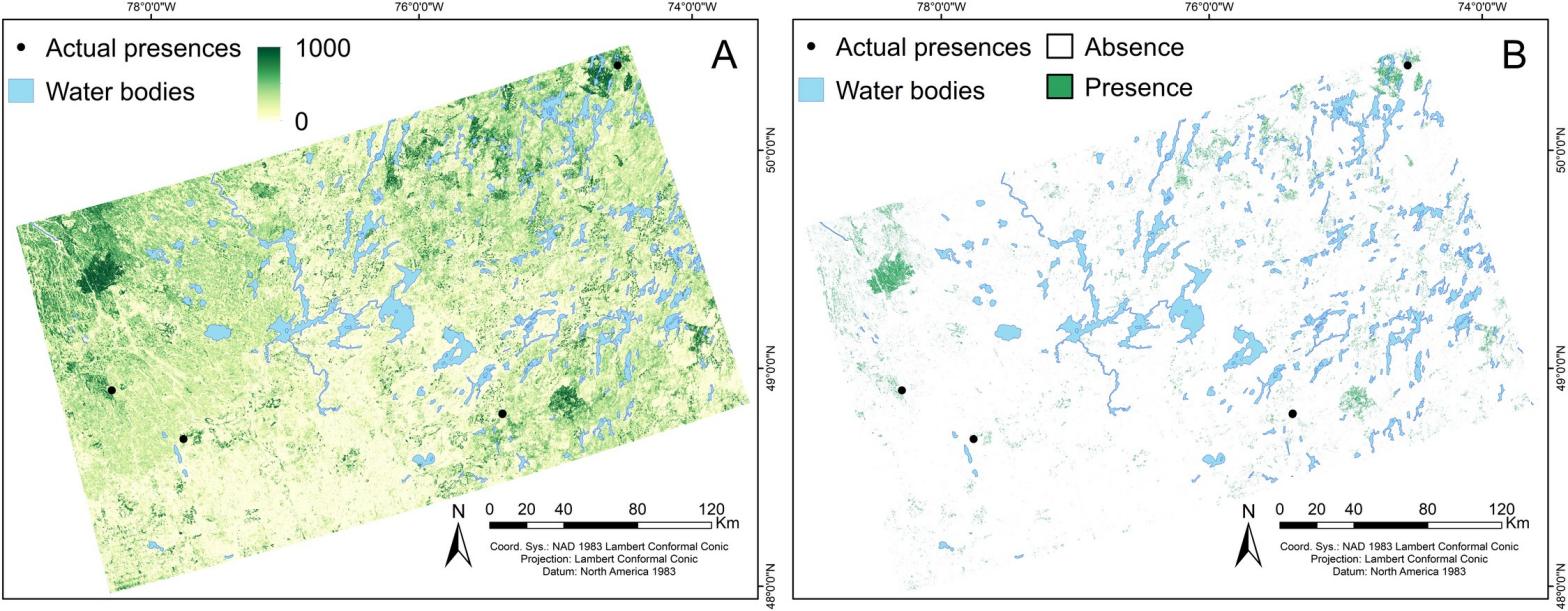
Example of (A) continuous and (B) binary predictive mapping of the moss *Trematodon ambiguus* (Hedw.) Hornsch. for the study area at 30 m spatial resolution.

ESMs were generated in R v.3.6.3 [74] using the *biomod2* package v.3.4.6 [75]. As we used presence-only data, 10,000 background points were randomly generated within the study area and used as pseudo-absences for all species. Presences and pseudo-absences were weighted equally for training the ESMs. The pairwise combinations of our 5 final predictors resulted in 10 candidate bivariate models per modeling technique (Maxent and RF) for each species. We used default settings of the *biomod2* package for computing Maxent and RF models. Predictive performance of each bivariate model was assessed via 10-fold cross-validation procedure, using 80% of the data to train the model and 20% for its validation. While we acknowledge that validation would be optimal using an external dataset, this is hardly available when dealing with rare species. The Somers’ D metric was used to identify and select bivariate models better than random (Somers’ D score > 0, i.e. AUC > 0.5). Maxent-ESMs and RF-ESMs were then performed using a weighted mean of predicted probabilities from their corresponding retained bivariate models based on their Somers’ D scores [20, 28]. The contribution of each bivariate model was thus proportional to its predictive accuracy. The final ESMs selected for each species was generated by weighted averaging predictions from Maxent-ESMs and RF-ESMs. Predictive performance of final ESMs was evaluated using the area under the receiver operating characteristic curve (AUC), and the true skills statistic (TSS). AUC is not dependent on a threshold and ranges from 0.5 for an uninformative model to 1 for a perfect fit model, while TSS ranges from -1 to 1 and was chosen instead of kappa because it is not affected by prevalence [76]. Since AUC and TSS values were highly correlated (Pearson r > 0.95), the results and discussion on models’ overall predictive performance will be based on the AUC statistics, following [28] and [76]. The statistic sensitivity was also calculated, which allows the assessment of the proportion of actual presences correctly predicted [77]. We computed sensitivity for those species whose final ESMs were better than random (AUC > 0.5). Besides of the continuous models (values 0–1000), we generate binary models (presence/absence) using the maximum training sensitivity plus specificity threshold, or TSS optimum (Fig 2; predictive mapping of the distribution of the target species is available in [78]). Finally, we mapped the richness patterns (species number) for total rare bryophyte species, as well as for rare species by guild, by stacking their binary predictions (presence/absence). Missing values associated with the predictions of the three species that included the VCF predictor in their final models were classified as absences before richness computation. We then compared the spatial richness patterns obtained here for rare species with those obtained recently for overall bryophyte species in a smaller region (28,436 km^2^) but fully included in our study area at the same spatial resolution (30 m) [18]. The comparison was performed for bryophytes as a whole (i.e. rare bryophyte richness versus overall bryophyte richness), and between homologous bryophyte guild pairs. This spatial correspondence analysis was carried out using Lee’s L statistic [79] through the *lee()* function from the *spdep* package v.1.1–5. [80]. Lee’s L statistic, in contrast to non-spatial bivariate association measures such as Pearson’s correlation coefficient, integrates and corrects for the spatial autocorrelation of each variable when computing the pixel-to-pixel spatial correlation [79]. Due to the high computational requirements to carry out this analysis, the 30 m pixels were previously averaged into 300 m pixels through the *aggregate()* function of the *raster* package v.3.4–5 [81]. Outputs of *lee()* function were centered at 0 and re-scaled to -1 and 1 to facilitate the interpretation of the results by subtracting the overall mean and dividing by the maximum value [82]. We then calculated, for each pixel, the quantile associated with its Lee’s L value using a Monte Carlo test with 999 simulations in order to identify significant positive (quantile >0.975) or negative (quantile <0.025) spatial associations.

### 2.4 Species traits characterization

Species traits can influence the accuracy and therefore the ability of SDMs to predict their occurrence [83, 84]. We evaluated the relationship between ESMs’ model performance, as measured by AUC, and rare species traits, namely substrate preference (six categories), reproduction mode (three categories), and spore size (maximum and minimum; S1 Table in S1 Appendix), as well as their interactions. This assessment was performed using a multiple linear regression through the *lm()* function from the *stats* package v.3.6.3 [74]. Relationships were considered significant at α = 0.05.

## 3. Results

### 3.1 ESMs’ predictive performance versus number of occurrences and bryophyte guilds

**Fig 3:**
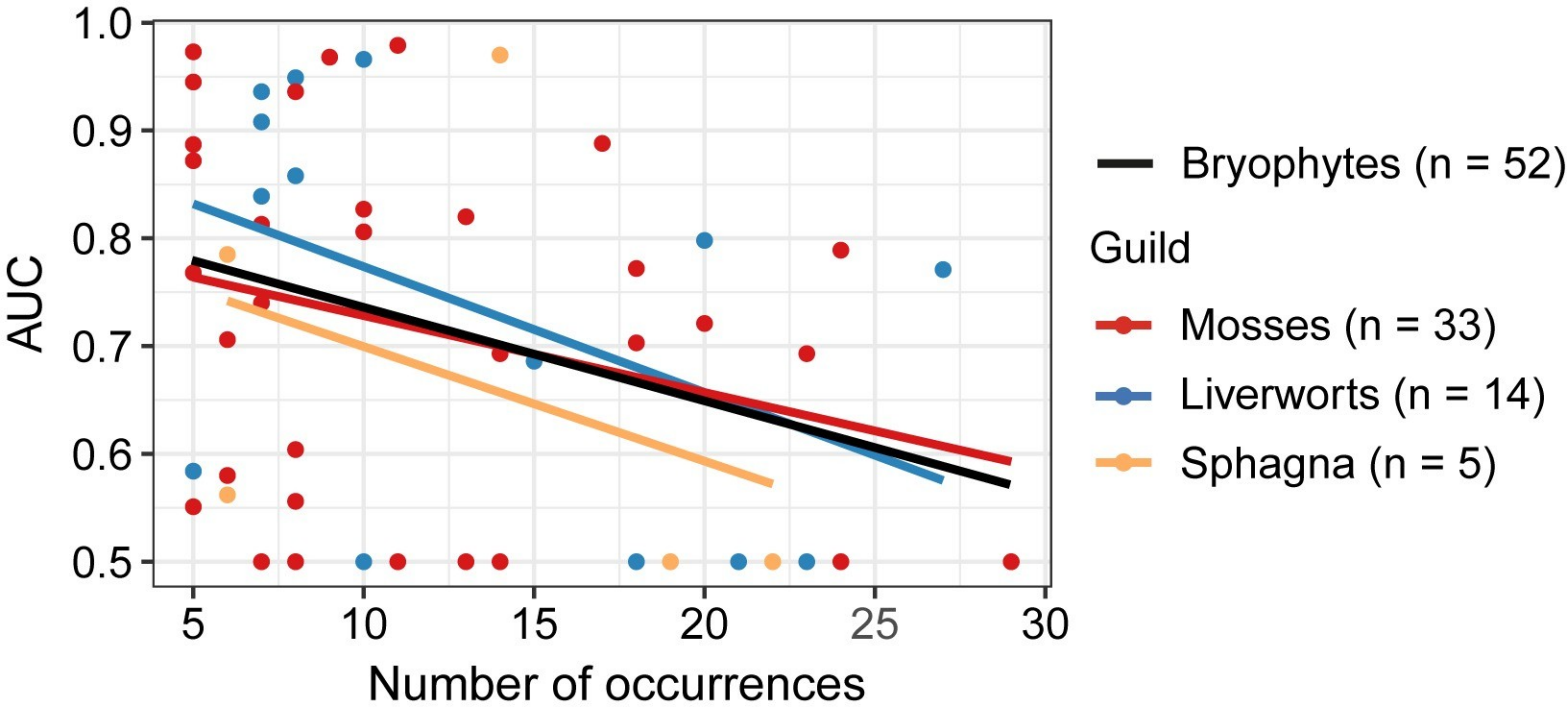
AUC values versus number of occurrences (Overall Pearson r = -0.34). Bryophyte guilds are indicated.

RS-based ESMs provided poor to excellent predictive accuracy for 38 of the 52 modeled rare species, with AUC values ranging from 0.551 to 0.979 and a mean AUC (mAUC) of 0.795 ± 0.132. Of these 38 species, 19 species were predicted with AUC values greater than 0.8, confirming our hypothesis that high ESMs-based prediction accuracy can be achieved for rare bryophyte species despite their low number of occurrences (<30). Sensitivity for these 38 species ranged from 0.8 to 1 with an average of 0.959 ± 0.063, indicating that actual presences were usually accurately predicted. Only predictions for 14 species were not better than random (AUC ≤ 0.5). Regarding our first specific objective, a negative correlation (Pearson r = -0.34) was found between the number of occurrences of the 52 target species and the predictive accuracy as measured by AUC. This negative correlation was also observed at the guild level (Fig 3).

To accomplish our second specific objective, we grouped the 52 modeled species by guild and found that predictive accuracy was similar for mosses (mAUC = 0. 715 ± 0.167) and liverworts (mAUC = 0.735 ± 0.185), and lower for sphagna (0.663 ± 0.208). No significant relationships were found between ESMs’ performance and rare species traits (or their interactions).

### 3.2 Richness patterns of rare bryophyte species

**Fig 4:**
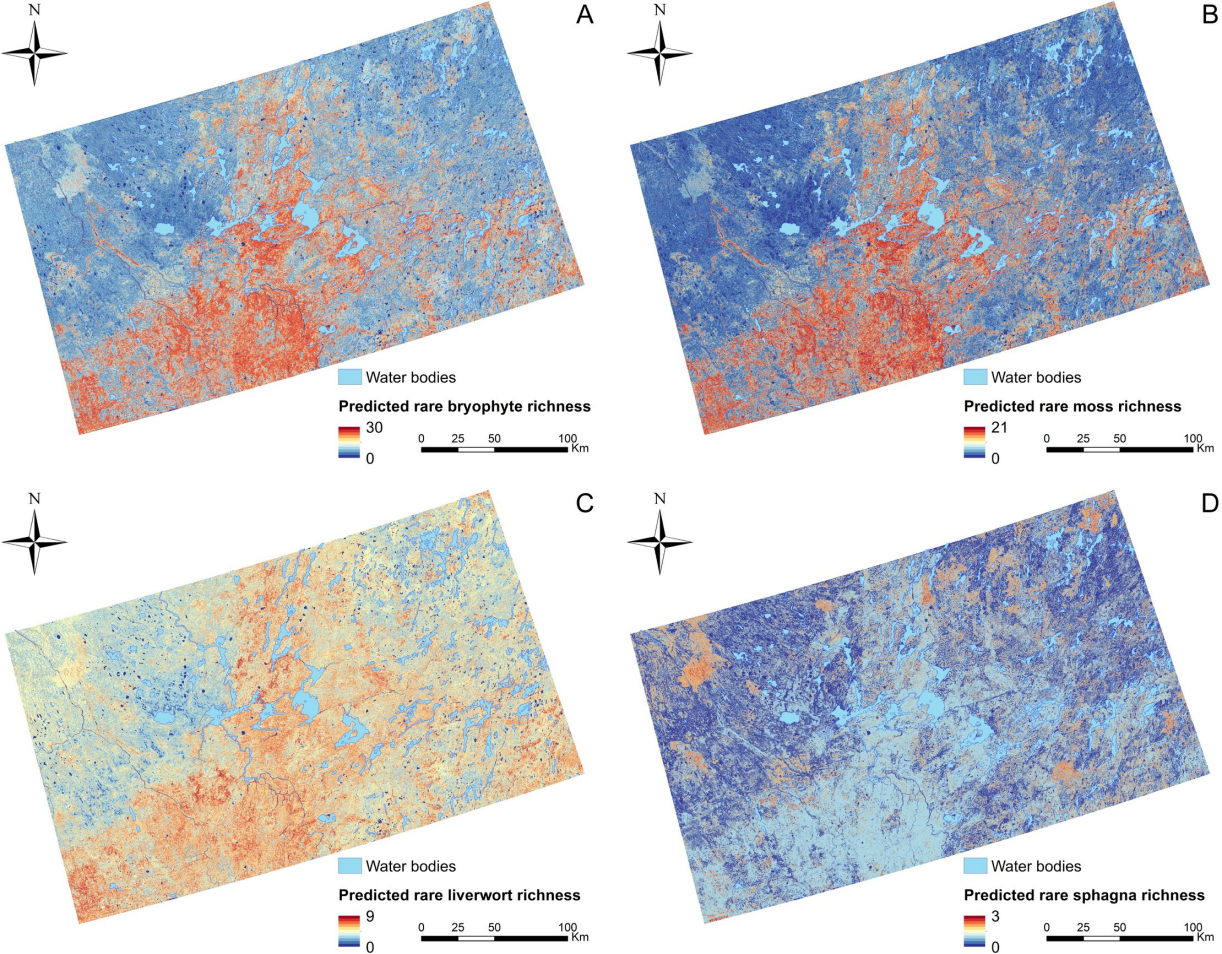
Mapping of (A) total rare bryophyte, (B) rare moss, (C) rare liverwort, and (D) rare sphagna richness (species number) for the study area at 30 m resolution. Computed from stacked predicted rare species distributions.

Predictive mapping of richness patterns of total rare bryophyte species and rare species at the guild level (mosses, liverworts and sphagna) are presented in Fig 4. Predicted richness values ranged from 0 to 30, 21, 9, and 3 species, respectively. The richness pattern of total rare bryophytes was largely structured by the similar richness patterns observed for rare mosses and liverworts, with high richness values mostly found towards the center and southwest of the study area. Conversely, rare sphagna species were concentrated in very specific areas mainly towards the north of the study area with two additional spots towards the southeast.

**Fig 5:**
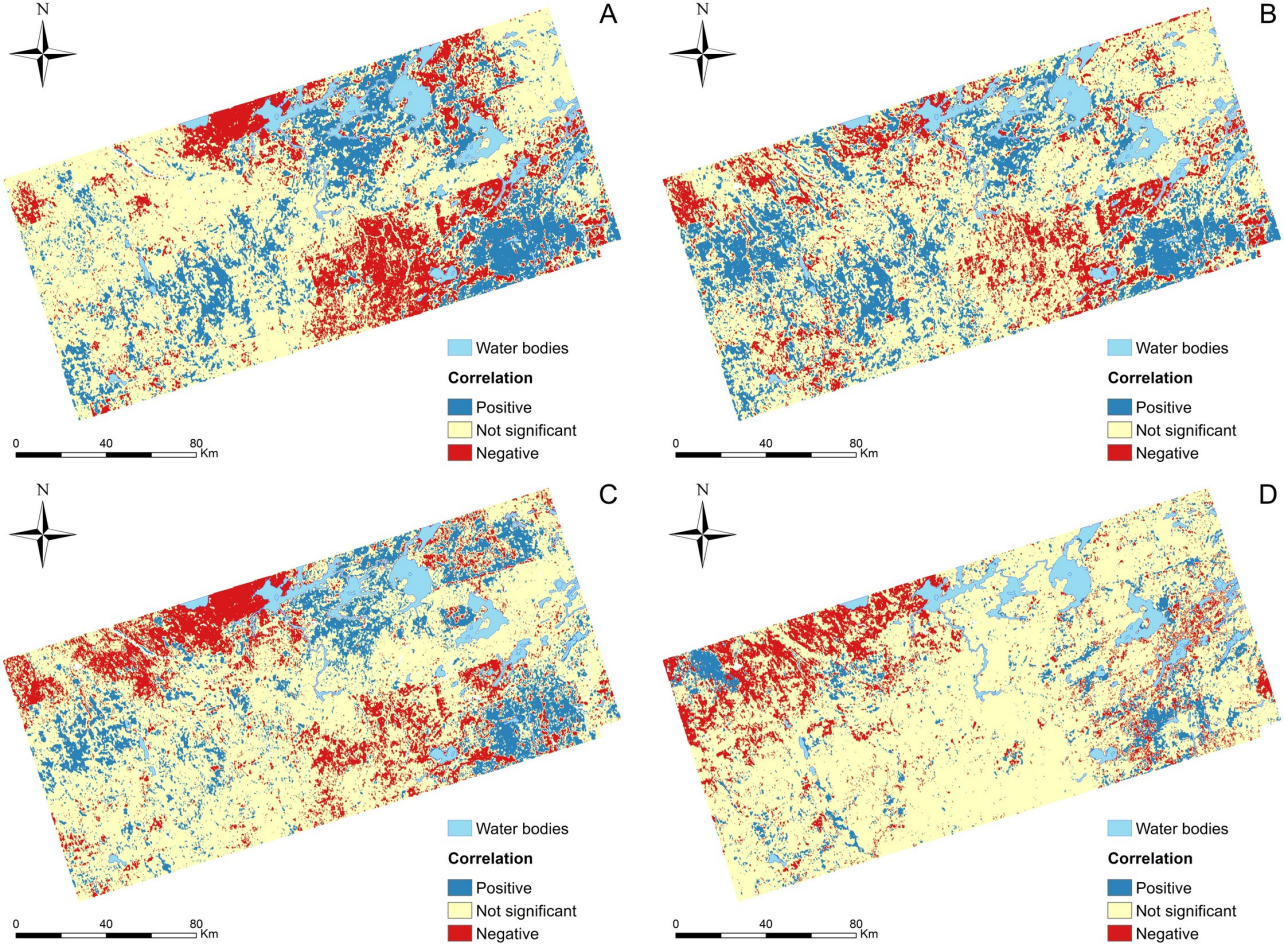
Correlation between rare and overall (A) bryophyte, (B) moss, (C) liverwort, and (D) sphagna species richness as measured by re-scaled Lee’s L statistic for the study area of [18] at 300 m spatial resolution. "Positive" (blue) and "Negative"(red) indicate significant positive (quantile >0.975) and negative (quantile <0.025) Lee’s L values derived from Monte Carlo test. Continuous values of the re-scaled Lee’s L statistic are shown in S1 Fig in S1 Appendix.

Regarding our third specific objective, the Lee’s L statistic identified areas of significant positive and negative spatial association between rare and overall species richness for the four homologous bryophyte group pairs (Fig 5). Large areas in which the spatial association between the two types of richness was not significant were also consistently observed across pairs.

## 4. Discussion

**Fig 6:**
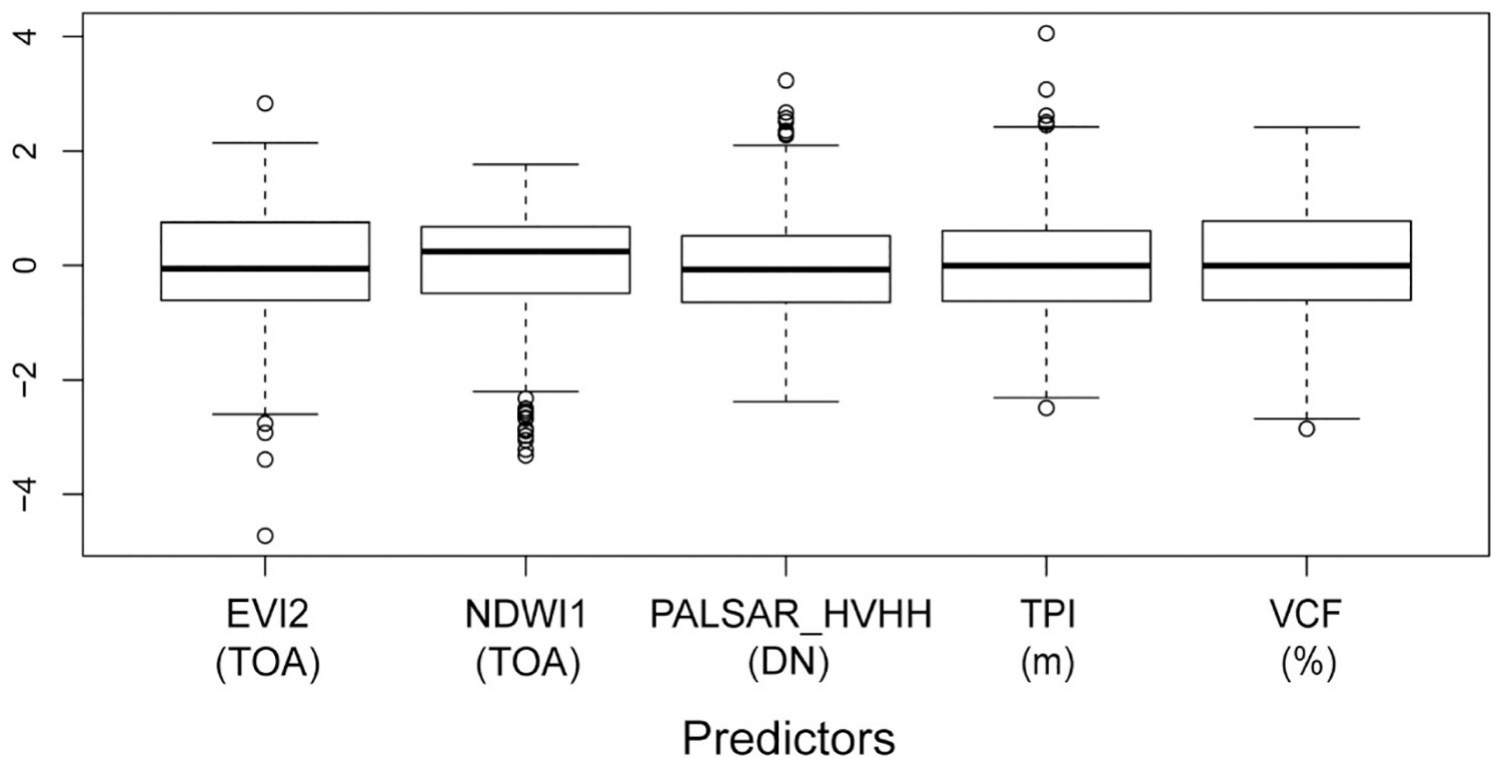
Boxplots of standardized uncorrelated predictors used for modeling. See Table 1 for predictor descriptions. Measurement units are indicated in parentheses. Unit abbrev.: DN, digital number; TOA, top-of-atmosphere reflectance.

Boreal regions are large areas lacking sharp environmental contrasts, as shown by the low variability of our predictors (Fig 6), and thus a habitat where obtaining high-performance SDMs can be challenging. Despite this, our ESMs provided reasonably accurate predictions for rare bryophytes using only 5 uncorrelated RS predictors. Specifically, RS-based ESMs provided poor to excellent predictive accuracy for 73% of the target species despite their very low number of occurrences. Indeed, 16 species with less than 10 occurrences showed an AUC > 0.7. In addition, the computation of the metric sensitivity allowed us to independently show the ability of our ESMs to accurately predict known presences, with high values for the 38 species modeled better than random. Therefore, the combination of RS data at 30 m spatial resolution and ESMs proved to be a powerful approach to predict the distribution of rare bryophyte species in Eastern Canadian boreal forests.

The negative relationship found between models’ predictive performance and the number of occurrences of all bryophytes, as well as at the guild level (Fig 3), illustrated the suitability of ESMs for predicting the distribution of very rare bryophyte species regardless of guild. This result agrees with those obtained in [28], who showed a higher predictive performance of ESMs for the rarest vascular plants. Regarding bryophyte species by guild, we consider that the lower overall predictive performance obtained for sphagna species compared to that of mosses and liverworts may be an artifact resulting from the low number of rare sphagna species modeled (n = 5). In fact, the occurrences of two of these five sphagna species were successfully predicted (AUC values of 0.76 and 0.97). However, we do not exclude the possibility that some ecologically meaningful variables that describe the habitat of these species, such as drainage class [18], were missing from our models.

In general, our results show that the development of SDMs from RS data allows not only to make predictions of rare species distribution at spatial scales relevant to ecological planning, but also to do so at a level of detail (30 m resolution) that can not be achieved using the traditionally used climatic variables at coarse resolutions (≥ 1 km). This is particularly important for inconspicuous species such as bryophytes, which interact with their environment at more local scales [85–87] and for which the use of coarse resolutions can result in a critical lose of information. Likewise, SDMs developed at coarse resolutions can overestimate species distribution [88] and greatly limits the practical utility of derived predictions to subsequently detect species in the field [89]. On the other hand, the wide variety of potentially relevant predictors for rare plants that can be derived from RS (related to vegetation, humidity, forest structure, topography, etc.) [90], can allow a more realistic approach to the environment-species relationship, which can be particularly useful for species with complex ecological niches. Thus, our methodology can play an important role in filling existing knowledge gaps on bryophyte distribution ranges, as well as their ecological preferences, in largely unexplored regions such as boreal forests [36].

The Identification of diversity hotspots has been one of the most used criteria in biodiversity conservation planning in order to locate areas of biological and ecological interest that should be prioritized by decision makers [91–93]. Conservation measures targeting these areas will be more effective if multiple components of biodiversity are spatially concentrated [92, 94, 95]. Specifically, both species richness and the presence of rare species have frequently been cited as the main criteria to select areas for conservation [96, 97], while many rare species might not be represented in species-rich areas [94]. Our study however revealed a spatial concordance between the richness of overall bryophyte species and that of their rare taxa in different regions of the study area (Fig 5). While more bryophyte biodiversity components could be subsequently evaluated, this result have important implications for Canadian conservation planning. We consider that the identification of areas harboring high level of both overall and rare bryophyte species diversity, as well as the development of informative tools that serve these purposes, is a significant and necessary step to promote the systematic integration of these species into conservation plans and programs [91]. Likewise, conservation planning targeting bryophytes and other inconspicuous taxa could further benefit from individual SDMs-based predictions as a basis for assessing their representation in nature reserve networks [98], to quantify the impact of land use changes on their distribution ranges [99], to inform assessments of their conservation status [100, 101], and to identify suitable areas for their recovery or reintroduction [102].

## 5. Conclusions

Our work demonstrates the ability for RS data to characterize the habitat of rare bryophyte species and predict their distribution patterns across the landscape. This study also reaffirms the effectiveness of ESMs in estimating rare plant distributions [20, 28], and highlights, for the first time, the suitability of this modeling approach for making predictions of inconspicuous rare species. We consider that our methods and results provide an important advance in the application of techniques focused on the study of bryophytes, with potential valuable applications for their management and conservation. In fact, although our study focuses on a particular taxonomic group, the combined use of ESMs and RS would lend useful results for other overlooked inconspicuous taxa lacking information on distribution, which would facilitate their integration in systematic conservation planning.

## Supporting information

S1 Table Species occurrence coordinates used for modeling. (XLSX)

S2 Table Standardized predictor values used for modeling. (XLSX)

S1 Appendix (DOCX)

## References

[1] Natural Resources Canada-Canadian Forest Service. Annual Report, The State of Canada’s Forests. 2017.

[2] Ficken CD, Cobbaert D, Rooney RC. Low extent but high impact of human land use on wetland flora across the boreal oil sands region. Sci. Total Environ. 2019; 693: 133647. doi: 10.1016/j.scitotenv.2019.133647 31635014

[3] Newmaster SG, Bell FW. The effects of silvicultural disturbances on cryptogam diversity in the boreal-mixedwood forest. Can. J. For. Res. 2002; 32(1): 38–51.

[4] Frego KA. Bryophytes as potential indicators of forest integrity. For. Ecol. Manage. 2007; 242(1): 65–75.

[5] Hylander K, Jonsson BG, Nilsson C. Evaluating buffer strips along boreal streams using bryophytes as indicators. Ecol. Appl. 2002; 12(3): 797–806.

[6] Vellak K, Ingerpuu N. Management effects on bryophytes in Estonian forests. Biodivers. Conserv. 2005; 14(13): 3255–3263.

[7] Möls T, Vellak K, Vellak A, Ingerpuu N. Global gradients in moss and vascular plant diversity. Biodivers. Conserv. 2013; 22(6): 1537–1551.

[8] Turetsky MR, Bond‐Lamberty B, Euskirchen E, Talbot J, Frolking S, McGuire AD, et al. The resilience and functional role of moss in boreal and arctic ecosystems. New Phytol. 2012; 196(1): 49–67. doi: 10.1111/j.1469-8137.2012.04254.x 22924403

[9] Bond-Lamberty B, Gower ST. Estimation of stand-level leaf area for boreal bryophytes. Oecologia 2007; 151(4): 584–592. doi: 10.1007/s00442-006-0619-5 17160690

[10] Turetsky MR. The role of bryophytes in carbon and nitrogen cycling. Bryologist 2003; 106(3): 395–409.

[11] Caners RT, Macdonald SE, Belland RJ. Bryophyte assemblage structure after partial harvesting in boreal mixedwood forest depends on residual canopy abundance and composition. For. Ecol. Manage. 2013; 289: 489–500.

[12] Boudreault C, Paquette M, Fenton NJ, Pothier D, Bergeron Y. Changes in bryophytes assemblages along a chronosequence in eastern boreal forest of Quebec. Can. J. For. Res. 2018; 48(7): 821–834.

[13] Rowntree JK, Pressel S, Ramsay MM, Sabovljevic A, Sabovljevic M. In vitro conservation of European bryophytes. In Vitro Cell. Dev. Biol. Plant 2011; 47(1): 55–64.

[14] Vanderpoorten A, Hallingbäck T. Conservation biology of bryophytes. Bryophyte Biol. 2009; 487–533.

[15] Austin MP, Margules CR. Assessing representativeness. In: Usher MB (ed.) Wildlife Conservation Evaluation. Chapman and Hall, London. 1986. pp. 45–67.

[16] Pimm SL, Jenkins CN, Abell R, Brooks TM, Gittleman JL, Joppa LN, et al. The biodiversity of species and their rates of extinction, distribution, and protection. Science 2014; 344(6187). 10.1126/science.1246752 24876501

[17] Rodrigues AS, Brooks TM. Shortcuts for biodiversity conservation planning: the effectiveness of surrogates. Annu. Rev. Ecol. Evol. Syst. 2007; 38: 713–737.

[18] Cerrejón C, Valeria O, Mansuy N, Barbé M, Fenton NJ. Predictive mapping of bryophyte richness patterns in boreal forests using species distribution models and remote sensing data. Ecol. Indic. 2020; 119: 106826.

[19] Delso A, Muñoz J, Fajardo J. Protected area networks do not represent unseen diversity. Sci. Rep. 2021; 11: 12275. doi: 10.1038/s41598-021-91651-z 34112867 PMC8192537

[20] Lomba A, Pellissier L, Randin C, Vicente J, Moreira F, Honrado J, et al. Overcoming the rare species modelling paradox: A novel hierarchical framework applied to an Iberian endemic plant. Biol. Conserv. 2010; 143(11): 2647–2657.

[21] Zhang P. Effective predictors of herbaceous plant diversity responses to changes in nutrient availability and herbivory. Doctoral dissertation, Utrecht University. 2019.

[22] Hortal J, de Bello F, Diniz-Filho JAF, Lewinsohn TM, Lobo JM, Ladle RJ. Seven shortfalls that beset large-scale knowledge of biodiversity. Annu. Rev. Ecol. Evol. Syst. 2015; 46: 523–549.

[23] Whittaker RJ, Araújo MB, Jepson P, Ladle RJ, Watson JE, Willis KJ. Conservation biogeography: assessment and prospect. Divers. Distrib. 2005; 11(1): 3–23.

[24] Guillera‐Arroita G, Lahoz‐Monfort JJ, Elith J, Gordon A, Kujala H, Lentini PE, et al. Is my species distribution model fit for purpose? Matching data and models to applications. Global Ecol. Biogeogr. 2015; 24(3): 276–292.

[25] Mateo RG, Felicisimo AM, Munoz J. Species distributions models: A synthetic revision. Rev. Chil. Hist. Nat. 2011; 84(2): 217–240.

[26] Guisan A, Graham CH, Elith J, Huettmann F, NCEAS Species Distribution Modelling Group. Sensitivity of predictive species distribution models to change in grain size. Divers. Distrib. 2007; 13(3): 332–340.

[27] Wisz MS, Hijmans RJ, Li J, Peterson AT, Graham CH, Guisan A, et al. Effects of sample size on the performance of species distribution models. Divers. Distrib. 2008; 14(5): 763–773.

[28] Breiner FT, Guisan A, Bergamini A, Nobis MP. Overcoming limitations of modelling rare species by using ensembles of small models. Methods Ecol. Evol. 2015; 6(10): 1210–1218.

[29] Vaughan IP, Ormerod SJ. The continuing challenges of testing species distribution models. J. Appl. Ecol. 2005; 42(4): 720–730.

[30] Amirkhiz RG, Dixon MD, Palmer JS, Swanson DL. Investigating niches and distribution of a rare species in a hierarchical framework: Virginia’s Warbler (Leiothlypis virginiae) at its northeastern range limit. Landsc. Ecol. 2021; 36(4): 1039–1054.

[31] He KS, Bradley BA, Cord AF, Rocchini D, Tuanmu MN, Schmidtlein S, et al. Will remote sensing shape the next generation of species distribution models?. Remote Sens. Ecol. Conserv. 2015; 1(1): 4–18.

[32] Turner W, Spector S, Gardiner N, Fladeland M, Sterling E, Steininger M. Remote sensing for biodiversity science and conservation. Trends Ecol. Evol. 2003; 18(6): 306–314.

[33] Jiang Y, De Bie CAJM, Wang T, Skidmore AK, Liu X, Song S, et al. Hyper‐temporal remote sensing helps in relating epiphyllous liverworts and evergreen forests. J. Veg. Sci. 2013; 24(2): 214–226.

[34] Saatchi S, Buermann W, Ter Steege H, Mori S, Smith TB. Modeling distribution of Amazonian tree species and diversity using remote sensing measurements. Remote Sens. Environ. 2008; 112(5): 2000–2017.

[35] Zimmermann NE, Edwards TC Jr, Moisen GG, Frescino TS, Blackard JA. Remote sensing‐based predictors improve distribution models of rare, early successional and broadleaf tree species in Utah. J. Appl. Ecol. 2007; 44(5): 1057–1067. doi: 10.1111/j.1365-2664.2007.01348.x 18642470 PMC2368764

[36] Barbé M, Dubois L, Faubert J, Lavoie M, Bergeron Y, Fenton NJ. Range Extensions of 35 Bryophyte Species in the Black Spruce–Feather Moss Forest of Western Quebec, Canada. Can. Field-Nat. 2018; 131(3): 258–269.

[37] Rabinowitz D. Seven forms of rarity. In: Synge H. (ed.) The biological aspects of rare plant conservation. Wiley. 1981. pp. 205–217.

[38] Faubert J, Lapointe M, Tardif B. Les bryophytes rares du Québec: espèces prioritaires pour la conservation. Centre de données sur le patrimoine naturel du Québec, Ministère du développement durable, de l’environnement et des parcs. 2010.

[39] Faubert J, Bastien DF, Gilbert H. Mise à jour de la publication Les bryophytes rares du Québec. Espèces prioritaires pour la conservation. Carnets de bryol. 2011; 1: 29–31.

[40] Faubert J, Gagnon J, Tremblay B, Couillard L. Mise à jour de la publication Les bryophytes rares du Québec. Espèces prioritaires pour la conservation. -2 –Carnets de bryol. 2012; 2: 53–56.

[41] Barbé M, Fenton NJ, Bergeron Y. Are post-fire residual forest patches refugia for boreal bryophyte species? Implications for ecosystem based management and conservation. Biodivers. Conserv. 2017; 26(4): 943–965.

[42] Castonguay, J. Dynamique des communautés de bryophytes dans la pessière à mousses de la forêt boréale: rôle des îlots de rétention après coupe, M.Sc. Thesis, Université du Québec à Montréal, Montreal (Quebec, Canada). 2016.

[43] Chaieb C, Fenton NJ, Lafleur B, Bergeron Y. Can we use forest inventory mapping as a coarse filter in ecosystem based management in the black spruce boreal forest? Forests 2015; 6(4): 1195–1207.

[44] Saucier, JP, Grondin, P, Robitaille, A, Bergeron, JF. Zones de végétation et domaines bioclimatiques du Québec. Publication No. 2003–3015. Ministère des Ressources naturelles, de la Faune et des Parcs (MRNFP), direction des inventaires forestiers. Quebec, Canada. 2003.

[45] Bergeron Y, Gauthier S, Flannigan M, Kafka V. Fire regimes at the transition between mixedwood and coniferous boreal forest in northwestern Quebec. Ecology 2004; 85(7): 1916–1932.

[46] Environment Canada. 2010. https://climate.weather.gc.ca/climate_normals/ (accessed 28 March 2019).

[47] Bergeron Y, Drapeau P, Gauthier S, Lecomte N. Using knowledge of natural disturbances to support sustainable forest management in the northern Clay Belt. For. Chron. 2007; 83(3): 326–337.

[48] Boudreault C, Bergeron Y, Gauthier S, Drapeau P. Bryophyte and lichen communities in mature to old-growth stands in eastern boreal forests of Canada. Can. J. For. Res. 2002; 32(6): 1080–1093.

[49] Newmaster SG, Belland RJ, Arsenault A, Vitt DH, Stephens TR. The ones we left behind: comparing plot sampling and floristic habitat sampling for estimating bryophyte diversity. Divers. Distrib. 2005; 11(1): 57–72.

[50] Hernandez PA, Graham CH, Master LL, Albert DL. The effect of sample size and species characteristics on performance of different species distribution modeling methods. Ecography 2006; 29(5): 773–785.

[51] Pearson RG, Raxworthy CJ, Nakamura M, Townsend Peterson A. Predicting species distributions from small numbers of occurrence records: a test case using cryptic geckos in Madagascar. J. Biogeogr. 2007; 34(1): 102–117.

[52] Spiers JA, Oatham MP, Rostant LV, Farrell AD. Applying species distribution modelling to improving conservation based decisions: a gap analysis of Trinidad and Tobago’s endemic vascular plants. Biodivers. Conserv. 2018; 27(11): 2931–2949.

[53] Couvreur JM, San Martin G, Sotiaux A. Factors affecting the presence and the diversity of bryophytes in the petrifying sources habitat (7220) in Wallonia and the Brussels-Capital Region, Belgium. Int. J. Agron. 2016; 2016.

[54] Jiang Y, Wang T, De Bie CAJM, Skidmore AK, Liu X, Song S, et al. Satellite-derived vegetation indices contribute significantly to the prediction of epiphyllous liverworts. Ecol. Indic. 2014; 38: 72–80.

[55] Raabe S, Müller J, Manthey M, Dürhammer O, Teuber U, Göttlein A, et al. Drivers of bryophyte diversity allow implications for forest management with a focus on climate change. For. Ecol. Manage. 2010; 260(11): 1956–1964.

[56] Gorelick N, Hancher M, Dixon M, Ilyushchenko S, Thau D, Moore R. Google Earth Engine: Planetary-scale geospatial analysis for everyone. Remote Sens. Environ. 2017; 202: 18–27.

[57] ESRI. ArcGIS Desktop. v. 10.5. Redlands, CA: Environmental Systems Research Institute. 2016.

[58] Franch B, Vermote E, Skakun S, Roger JC, Masek J, Ju J, et al. A method for Landsat and Sentinel 2 (HLS) BRDF normalization. Remote Sens. 2019; 11(6): 632.

[59] Jiang Z, Huete AR, Kim Y, Didan K. 2-band enhanced vegetation index without a blue band and its application to AVHRR data. In: Remote Sensing and Modeling of Ecosystems for Sustainability IV. International Society for Optics and Photonics. 2007; 6679: p. 667905.

[60] Sexton JO, Song XP, Feng M, Noojipady P, Anand A, Huang C, et al. Global, 30-m resolution continuous fields of tree cover: Landsat-based rescaling of MODIS vegetation continuous fields with lidar-based estimates of error. Int. J. Digital Earth 2013; 6(5): 427–448.

[61] Mansuy N, Valeria O, Laamrani A, Fenton NJ, Guindon L, Bergeron Y, et al. Digital mapping of paludification in soils under black spruce forests of eastern Canada. Geoderma Reg. 2018; 15: e00194. 10.1016/j.dib.2018.11.131 PMC6288461 30560164

[62] Gao BC. NDWI—A normalized difference water index for remote sensing of vegetation liquid water from space. Remote Sens. Environ. 1996; 58(3): 257–266.

[63] Bartels SF, Caners RT, Ogilvie J, White B, Macdonald SE. Relating bryophyte assemblages to a remotely sensed depth-to-water index in boreal forests. Front. Plant Sci. 2018; 9: 858. doi: 10.3389/fpls.2018.00858 29988528 PMC6026670

[64] Gignac LD. Bryophytes as indicators of climate change. Bryologist 2001; 104(3): 410–420.

[65] Bennie J, Huntley B, Wiltshire A, Hill MO, Baxter R. Slope, aspect and climate: spatially explicit and implicit models of topographic microclimate in chalk grassland. Ecol. Modell. 2008; 216(1): 47–59.

[66] Moreira EF, Santos RLDS, Silveira MS, Boscolo D, Neves ELD, Viana BF. Influence of landscape structure on Euglossini composition in open vegetation environments. Biota Neotrop. 2017; 17(1).

[67] Townsend J, DiMiceli D—University of Maryland and MODAPS SIPS—NASA. MOD44B MODIS/Terra Vegetation Continuous Fields Yearly L3 Global 500m SIN Grid. NASA LP DAAC. 2015. 10.5067/MODIS/MOD44B.006.

[68] Roy PS, Sharma KP, Jain A. Stratification of density in dry deciduous forest using satellite remote sensing digital data—An approach based on spectral indices. J. Biosci. 1996; 21(5): 723–734.

[69] Phillips SJ, Anderson RP, Schapire RE. Maximum entropy modeling of species geographic distributions. Ecol. Modell. 2006; 190(3–4): 231–259.

[70] Breiman L. Random forests. Mach. Learn. 2001; 45: 5–32.

[71] Pouteau R, Meyer JY, Taputuarai R, Stoll B. Support vector machines to map rare and endangered native plants in Pacific islands forests. Ecol. Inf. 2012; 9: 37–46.

[72] Williams JN, Seo C, Thorne J, Nelson JK, Erwin S, O’Brien JM, et al. Using species distribution models to predict new occurrences for rare plants. Divers. Distrib. 2009; 15(4): 565–576.

[73] Liaw A, Wiener M. The randomforest package. R news 2002; 2: 18–22.

[74] R Development Core Team. R: A Language and Environment for Statistical Computing. Version 3.6.3. R Foundation for Statistical Computing, Vienna, Austria. 2020.

[75] Thuiller W, Georges D, Engler R, Breiner F. biomod2: Ensemble platform for species distribution modeling. R package version 3.4.6. 2020.

[76] Allouche O, Tsoar A, Kadmon R. Assessing the accuracy of species distribution models: prevalence, kappa and the true skill statistic (TSS). J. Appl. Ecol. 2006; 43(6): 1223–1232.

[77] Fawcett T. ROC graphs: Notes and practical considerations for researchers. Mach. Learn. 2004; 31(1): 1–38.

[78] Cerrejón C, Valeria O, Muñoz J, Fenton NJ. Mapping of rare bryophyte species distribution. Mendeley Data 2021; V3. https://data.mendeley.com/datasets/9mtcx3jnxz/2" https://data.mendeley.com/datasets/9mtcx3jnxz/3. doi: 10.17632/9mtcx3jnxz.3

[79] Lee SI. Developing a bivariate spatial association measure: an integration of Pearson’s r and Moran’s I. J. Geogr. Syst. 2001; 3(4): 369–385.

[80] Bivand R, Gómez-Rubio V, Rue H. Spatial data analysis with R-INLA with some extensions. American Statistical Association. 2015.

[81] Hijmans, R.J. raster: Geographic Data Analysis and Modeling. R package version 3.4–5. 2020. https://CRAN.R-project.org/package=raster.

[82] Mateo RG, Broennimann O, Normand S, Petitpierre B, Araújo MB, Svenning JC, et al. The mossy north: an inverse latitudinal diversity gradient in European bryophytes. Sci. Rep. 2016; 6(1): 1–9. doi: 10.1038/s41598-016-0001-8 27151094 PMC4858760

[83] Chefaoui RM, Lobo JM, Hortal J. Effects of species’ traits and data characteristics on distribution models of threatened invertebrates. Anim. Biodivers. Conserv. 2011; 34(2): 229–247.

[84] McCune JL, Rosner‐Katz H, Bennett JR, Schuster R, Kharouba HM. Do traits of plant species predict the efficacy of species distribution models for finding new occurrences?. Ecol. Evol. 2020; 10(11): 5001–5014. doi: 10.1002/ece3.6254 32551077 PMC7297770

[85] Cole HA, Newmaster SG, Bell FW, Pitt D, Stinson A. Influence of microhabitat on bryophyte diversity in Ontario mixedwood boreal forest. Can. J. For. Res. 2008; 38(7): 1867–1876.

[86] Hespanhol H, Séneca A, Figueira R, Sérgio C. Microhabitat effects on bryophyte species richness and community distribution on exposed rock outcrops in Portugal. Plant Ecolog. Divers. 2011; 4(2–3): 251–264.

[87] Pócs T. Epiphyllous liverwort diversity at worldwide level and its threat and conservation. An. Inst. Biol. Ser. Bot. 1996; 67(001).

[88] Lawler JJ, Wiersma YF, Huettmann F. Using species distribution models for conservation planning and ecological forecasting. In: Predictive species and habitat modeling in landscape ecology. Springer, New York. 2011. pp. 271–290.

[89] Guisan A, Broennimann O, Engler R., Vust M., Yoccoz NG, Lehmann A, et al. Using niche‐based models to improve the sampling of rare species. Conserv. Biol. 2006; 20(2): 501–511. doi: 10.1111/j.1523-1739.2006.00354.x 16903111

[90] Cerrejón C, Valeria O, Marchand P, Caners RT, Fenton NJ. No place to hide: Rare plant detection through remote sensing. Divers. Distrib. 2021.

[91] Hespanhol H, Cezón K, Felicísimo ÁM, Munoz J, Mateo RG. How to describe species richness patterns for bryophyte conservation?. Ecol. Evol. 2015; 5(23): 5443–5455. doi: 10.1002/ece3.1796 27069596 PMC4813098

[92] Myers N, Mittermeier RA, Mittermeier CG, Da Fonseca GA, Kent J. Biodiversity hotspots for conservation priorities. Nature 2000; 403(6772): 853–858. doi: 10.1038/35002501 10706275

[93] Prendergast JR, Quinn RM, Lawton JH. The gaps between theory and practice in selecting nature reserves. Conserv. Biol. 1999; 13(3): 484–492.

[94] Prendergast JR, Quinn RM, Lawton JH, Eversham BC, Gibbons DW. Rare species, the coincidence of diversity hotspots and conservation strategies. Nature 1993; 365(6444): 335–337.

[95] Ricketts TH, Dinerstein E, Boucher T, Brooks TM, Butchart SH, Hoffmann M, et al. Pinpointing and preventing imminent extinctions. Proc. Natl. Acad. Sci. 2005; 102(51): 18497–18501. doi: 10.1073/pnas.0509060102 16344485 PMC1311739

[96] Scott JM, Davis F, Csuti B, Noss R, Butterfield B, Groves C, et al. Gap analysis: a geographic approach to protection of biological diversity. Wildl. Monogr. 1993; 3–41.

[97] Usher MB. Wildlife conservation evaluation: attributes, criteria and values. In: Wildlife conservation evaluation. Springer, Dordrecht. 1986. pp. 3–44.

[98] Margules CR, Stein JL. Patterns in the distributions of species and the selection of nature reserves: an example from Eucalyptus forests in south-eastern New South Wales. Biol. Conserv. 1989; 50(1–4): 219–238.

[99] Thomas CD, Cameron A, Green RE, Bakkenes M, Beaumont LJ, Collingham YC, et al. Extinction risk from climate change. Nature 2004; 427(6970): 145–148. doi: 10.1038/nature02121 14712274

[100] Sousa-Silva R, Alves P, Honrado J, Lomba A. Improving the assessment and reporting on rare and endangered species through species distribution models. Global Ecol. Conserv. 2014; 2: 226–237.

[101] Syfert MM, Joppa L, Smith MJ, Coomes DA, Bachman SP, Brummitt NA. Using species distribution models to inform IUCN Red List assessments. Biol. Conserv. 2014; 177: 174–184.

[102] Pearce J, Lindenmayer D. Bioclimatic analysis to enhance reintroduction biology of the endangered helmeted honeyeater (Lichenostomus melanops cassidix) in southeastern Australia. Restor. Ecol. 1998; 6(3): 238–243.

