# Supplementary material for: Small but visible: Predicting rare bryophyte distribution and richness patterns using remote sensing-based ensembles of small models
Source: PLoS One. 2022 Jan 6;17(1):e0260543. doi: 10.1371/journal.pone.0260543 (PMC8735603; doi:10.1371/journal.pone.0260543)
Supplement: S1 Appendix — (DOCX) [file pone.0260543.s003.docx]

**S1 Table.** Modeled rare bryophyte species (n = 52) indicating the number of available occurrences as well as the predictive performance of ESMs as measured by AUC, TSS and Sensitivity. Bryophyte species traits, namely substrate preference, reproduction mode, and spore size are also included.

| **Species** | **# of occurrences** | **AUC** | **TSS** | **Sensitivity** | **Substrate preference** | **Reproduction mode** | **Spore size (µm)** |
| --- | --- | --- | --- | --- | --- | --- | --- |
| **Liverworts (n = 14)** |  |  |  |  |  |  |  |
| *Anastrophyllum minutum* | 8 | 0.858 | 0.767 | 0.8 | R | V | 12-14 |
| *Barbilophozia attenuata* | 23 | 0.5 | 0 | 0 | G | V | 10-14 |
| *Bazzania trilobata* | 7 | 0.936 | 0.933 | 1 | T | NA | 12-17 |
| *Calypogeia integristipula* | 7 | 0.839 | 0.834 | 1 | G | V | 10-13 |
| *Calypogeia suecica* | 5 | 0.584 | 0.384 | 1 | EO | V | 9-11 |
| *Cephaloziella elachista* | 15 | 0.686 | 0.494 | 1 | G | S | 9-11 |
| *Cephaloziella spinigera* | 10 | 0.966 | 0.962 | 0.95 | G | S | 7-10 |
| *Chiloscyphus coadunatus* | 21 | 0.5 | 0 | 0 | G | S | 15-20 |
| *Gymnocolea inflata* | 10 | 0.5 | 0 | 0 | G | VS | 12-18 |
| *Jungermannia leiantha* | 18 | 0.5 | 0 | 0 | G | S | 12-15 |
| *Lophozia ascendens* | 20 | 0.798 | 0.657 | 0.975 | EO | V | 9.5-10.5 |
| *Lophozia bicrenata* | 8 | 0.949 | 0.93 | 0.9 | G | VS | 12-16 |
| *Plagiochila porelloides* | 7 | 0.908 | 0.705 | 1 | G | S | 14-20 |
| *Tritomaria exsectiformis* | 27 | 0.771 | 0.523 | 0.84 | G | V | 9-12 |
| **Mosses (n = 33)** |  |  |  |  |  |  |  |
| *Amblystegium serpens* | 17 | 0.888 | 0.842 | 0.8668 | G | S | 8-15 |
| *Brachythecium erythrorrhizon* | 5 | 0.973 | 0.968 | 1 | R | S | 14-20 |
| *Brachythecium populeum* | 5 | 0.872 | 0.871 | 1 | G | S | 12-20 |
| *Brachythecium rutabulum* | 18 | 0.772 | 0.734 | 0.975 | G | S | 12-18 |
| *Brachythecium velutinum* | 20 | 0.721 | 0.487 | 0.8 | G | S | 13-16 |
| *Breidleria pratensis* | 7 | 0.5 | 0 | 0 | G | S | 10-13 |
| *Brotherella recurvans* | 14 | 0.693 | 0.496 | 0.8668 | G | S | 13-18 |
| *Bryum caespiticium* | 5 | 0.887 | 0.882 | 1 | T | S | 10-18 |
| *Callicladium haldanianum* | 23 | 0.693 | 0.449 | 1 | G | S | 10-18 |
| *Calliergon richardsonii* | 6 | 0.706 | 0.555 | 1 | P | S | 17-31 |
| *Campyliadelphus chrysophyllus* | 7 | 0.813 | 0.81 | 1 | G | S | 14-14 |
| *Campylophyllum hispidulum* | 13 | 0.5 | 0 | 0 | G | S | 9-13 |
| *Campylium stellatum* | 5 | 0.551 | 0.538 | 1 | P | S | 12-18 |
| *Drepanocladus aduncus* | 13 | 0.82 | 0.646 | 0.9667 | P | S | 16-16 |
| *Hygroamblystegium varium* | 8 | 0.5 | 0 | 0 | G | S | 10-16 |
| *Isopterygiopsis muelleriana* | 7 | 0.74 | 0.739 | 1 | R | VS | 8-12 |
| *Leptodictyum riparium* | 8 | 0.556 | 0.323 | 0.9 | G | S | 12-16 |
| *Mnium spinulosum* | 10 | 0.827 | 0.718 | 1 | G | S | 16-24 |
| *Plagiomnium cuspidatum* | 10 | 0.806 | 0.703 | 0.9 | G | S | 18-31 |
| *Plagiomnium drummondii* | 5 | 0.945 | 0.943 | 1 | T | S | 18-25 |
| *Plagiomnium medium* | 8 | 0.604 | 0.415 | 0.9 | T | S | 20-36 |
| *Plagiothecium denticulatum* | 29 | 0.5 | 0 | 0 | EF | S | 9-13 |
| *Platygyrium repens* | 18 | 0.703 | 0.623 | 0.85 | G | V | 13-18 |
| *Platydictya subtilis* | 6 | 0.58 | 0.527 | 1 | EO | S | 9-13 |
| *Pogonatum dentatum* | 8 | 0.936 | 0.892 | 1 | G | S | 18-24 |
| *Polytrichastrum longisetum* | 9 | 0.968 | 0.95 | 1 | P | S | 18-28 |
| *Polytrichastrum pallidisetum* | 5 | 0.768 | 0.761 | 1 | G | S | 12-16 |
| *Rhizomnium pseudopunctatum* | 24 | 0.5 | 0 | 0 | T | S | 40-50 |
| *Rhizomnium punctatum* | 11 | 0.5 | 0 | 0 | G | S | 29-41 |
| *Sarmentypnum exannulatum* | 19 | 0.5 | 0 | 0 | P | S | 16-20 |
| *Tomentypnum falcifolium* | 24 | 0.789 | 0.578 | 0.98 | P | S | NA |
| *Tomentypnum nitens* | 14 | 0.5 | 0 | 0 | P | S | 16-20 |
| *Trematodon ambiguus* | 11 | 0.979 | 0.973 | 1 | T | S | 30-36 |
| **Sphagna (n = 5)** |  |  |  |  |  |  |  |
| *Sphagnum cuspidatum* | 6 | 0.785 | 0.743 | 1 | P | S | 29-38 |
| *Sphagnum pulchrum* | 6 | 0.562 | 0.517 | 1 | P | S | 25-28 |
| *Sphagnum squarrosum* | 19 | 0.5 | 0 | 0 | G | S | 17-30 |
| *Sphagnum subtile* | 22 | 0.5 | 0 | 0 | T | S | 19-29 |
| *Sphagnum tenerum* | 14 | 0.97 | 0.937 | 0.9667 | P | S | 22-25 |

Substrate preference abbrev.: EF, facultative epixylic; EO, obligate epixylic; G, generalist; P, peatland; R, rock; T, terricolous. Reproduction mode abbrev.: S, sexual; V, vegetative; VS, vegetative and sexual. Information on bryophyte species traits was found in Faubert (2012, 2013, 2014), Boudreault et al. (2018), Barbé et al. (2017), Crum & Anderson (1981), BFNA (http://www.efloras.org/flora_page.aspx?flora_id=50), BRYOATT (Hill et al., 2007), and based on personal experience (laboratory of N.J. Fenton).


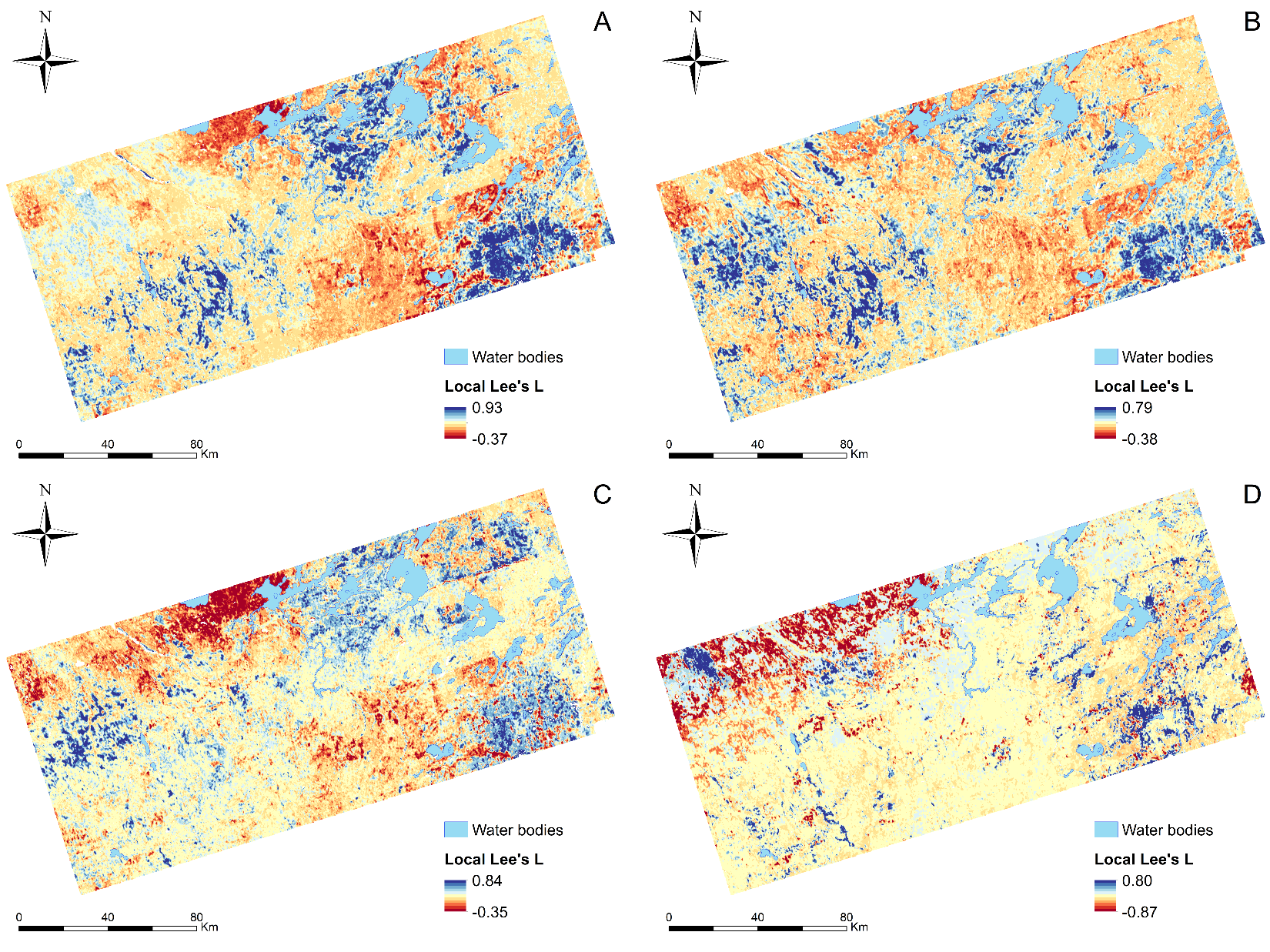
**S1 Fig.** Re-scaled Lee’s L bivariate spatial association between rare and overall (A) bryophyte, (B) moss, (C) liverwort, and (D) sphagna species richness for the study area of Cerrejón et al. (2020) at 300 m spatial resolution.
